# Conservation Challenges and Opportunities for *Fokienia hodginsii* in the Wuyi Mountains Under Climate Change and Human Influence

**DOI:** 10.1002/ece3.72887

**Published:** 2026-01-07

**Authors:** Dawei Luo, Tongli Wang, Jiejie Sun, Xiali Guo, Mingliang Peng, Hongxi Shen, Jing Qian

**Affiliations:** ^1^ Sanming University Sanming Fujian China; ^2^ Department of Forest and Conservation Sciences, Faculty of Forestry University of British Columbia Vancouver British Columbia Canada; ^3^ Jiangmen Laboratory of Carbon Science and Technology Hong Kong University of Science and Technology (Guangzhou) Jiangmen Guangdong China; ^4^ Guangxi Key Laboratory of Forest Ecology and Conservation College of Forestry Guangxi University Nanning China; ^5^ Sanyuan Forestry Survey and Planning Design Team Sanming Fujian China; ^6^ Huaqiao University Xiamen Fujian China

**Keywords:** human footprint, model comparison, predictive modeling, soil conditions, species distribution

## Abstract

*Fokienia hodginsii* (Dunn) A. Henry & H. H. Thomas, as an evergreen Tertiary relic conifer species of great ornamental, medical, and ecological value, has not been fully explored in terms of its risk associated with distribution under climate change scenarios. The Wuyi Mountains region is of exceptional ecological significance and provides important habitats for *F. hodginsii*. We compared four species distribution models (SDMs): Maximum Entropy Model (MaxEnt), random forest (RF), boosted regression tree (BRT), and generalized linear model (GLM) using climate variables, alongside soil variables and human footprint index, and used the best to make a comprehensive assessment of *F. hodginsii*'s environmental suitability under shared socioeconomic pathways (SSPs) 126 and 585. Our results indicate that MaxEnt model provided the best discriminative power and prediction accuracy in species distribution predictions, with Area Under Curve (AUC) value of 0.973, True Skill Statistic (TSS) value of 0.704, and Kappa of 0.395. We found that climate variables played the dominant role in shaping the distribution of *F. hodginsii* and accounted for 90.9% of the permutation importance. Furthermore, an overall trend of shrinking distribution was predicted for *F. hodginsii,* and it would face a huge loss of 97.6% suitable habitat under the scenario of SSP585. These findings indicate a potential loss of economic and ecological value of *F. hodginsii*, highlighting the risks posed to forest ecosystems in the Wuyi Mountains and underscoring the need for comprehensive conservation strategies to protect the species along with the economic benefits it provides.

## Graphical abstract

We assessed current and future habitat suitability for the vulnerable conifer Fokienia hodginsii across the Wuyi Mountains using climate, soil, and human footprint data, comparing four species distribution models and selecting MaxEnt as best. Climate—especially temperature ranges—dominated suitability, while soil and human influence were secondary. CMIP6 ensemble projections (SSP126/585) indicate severe range contraction, with up to ~97.6% habitat loss by the 2090s under SSP585, underscoring the need for targeted conservation.

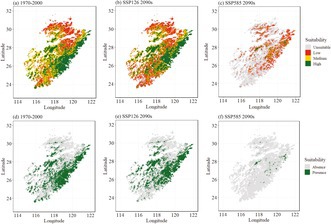

## 1 Introduction

The effects of climate change on species distribution are broadly observed. As species tend to retain their niche and related ancestral traits (Wiens and Graham 2005), the rapid climate change poses significant challenges to forest ecosystems and leads to potential mismatch between historical suitable habitat and future climate conditions. Forest species once well‐suited to their climate may face issues related to health and productivity, and consequent change in spatial distribution (Dai et al. 2016; Wan et al. 2018; Wang et al. 2017; Scheffers et al. 2016). Normally, the increasing temperature pushes the forest species distribution northward in latitude and upward in elevation in the Northern hemisphere, leaving forest ecosystems at the trailing edge potentially replaced by shrubs and grassland (Chen et al. 2011; Lenoir et al. 2008). To what extent climate change may affect the species distribution is dependent on the degree of climate change, the intensity of local adaptation, and the potential for migration (DeMarche et al. 2019). Thus, understanding the quantitative relationship between climate conditions and habitat suitability is a prerequisite for projecting the distribution in the future and planning species conservation strategies.

In addition to climate variables, human activities, and soil properties significantly influence tree species distribution. Human activities, such as forest management, construction, agriculture, and tourism, have direct impacts on tree species distribution by altering composition and structure (Fuller et al. 1998; Sun, Qiu, et al. 2020), especially for forest ecosystems near human settlements, where the intensity of human activities directly affects forest distribution and can lead to habitat loss. Some forestry activities, such as reforestation through assisted migration, aim to preserve distribution areas by planting populations that are climatically adapted to future conditions (Xu and Prescott 2024). Consequently, human activity intensity is a critical factor in forest conservation planning (Martinuzzi et al. 2021). Soil properties also play an important role in shaping suitable habitats. Soil condition is essential for the survival of plants, as soil texture, soil depth, composition, and nutrient availability can substantially affect water availability and nutrient uptake for tree growth (Binkley and Fisher 2019). Understanding the intricate relationships between soil properties and plant health is also necessary for habitat suitability modeling in the context of climate change (Feng et al. 2020; Sun et al. 2021).

*Fokienia hodginsii* (Dunn) A. Henry & H. H. Thomas is an evergreen Tertiary relic coniferous tree species of the monotypic genus *Fokienia*, and is primarily found in the warm, moist montane forests in Southeast Asia at elevations between 1000 and 1800 m (Zheng and Fu 1978). *Fokienia hodginsii* can reach heights of 20–30 m with a trunk diameter exceeding 1 m, providing excellent ornamental, medical, and ecological values (Huang et al. 2013). *F. hodginsii* is under increasing environmental stress as a result of climate change, such as decreasing regeneration, stand density, and growth rate (Dao and Hölscher 2017; Su et al. 2017). In addition, human activities such as selective logging for its high‐value timber and the conversion of mid‐elevation forests to tea and bamboo plantations were also major stresses for *F. hodginsii*. Therefore, it is also classified as vulnerable by the International Union for Conservation of Nature Red List and a second‐class national protected wild plant in China (https://www.gov.cn/zhengce/zhengceku/2021‐09/09/content_5636409.htm). Hence, the conservation of *F. hodginsii* is of great necessity and requires the understanding of species distribution in response to the changing climate. The current research on the climate adaptation of *F. hodginsii* has primarily focused on its growth response to historical climate variables (Sano et al. 2009; Su et al. 2017; Xu et al. 2011) and on estimating its potential distribution under present climatic conditions (Dan‐Qi et al. 2020). However, studies addressing the projected distribution of *F. hodginsii* under future climate change scenarios remain limited (Weng et al. 2025).

Species distribution models (SDMs) are commonly used tools in understanding environmental impacts on distribution dynamics through habitat suitability assessment based on local adaptation theory (DeMarche et al. 2019; Charney et al. 2021). Through quantifying the effects from environmental variation on species occurrence probability, they are widely applied to assess the potential impacts of climate change and land use (Zhao et al. 2014; Dyderski et al. 2018; Journé et al. 2020), evaluate potential risk of invasive species (Zhao et al. 2014), select sites for natural conservation (Li et al. 2021) and reforestation (Zhao et al. 2023; Zhao et al. 2024; Zhao and Wang 2023). Among the diverse algorithms, the Maximum Entropy model (MaxEnt) (Phillips et al. 2006; Phillips and Dudík 2008), random forest (RF) (Breiman 2001), generalized linear models (GLM) (Dobson and Barnett 2018), and boosted regression trees (BRT) (Yu et al. 2020) have gained popularity because they deliver high predictive accuracy while accommodating different data types (presence–absence or presence‐only). Adopting a multimodel framework and selecting the algorithm with the best cross‐validated performance is increasingly considered a robust approach for ecological forecasting and management recommendations (Zhao et al. 2023; Aierken et al. 2024).

Taking the Wuyi Mountains as a case study, we (1) calibrated MaxEnt, RF, BRT, and GLM models to species occurrence data of *F. hodginsii* combined with climate, soil data, and human footprint index as the explanatory variables, and selected the best performing model to (1) identify the driving factors for distribution of *F. hodginsii*; and (2) assess the impacts of climate change on the distribution of *F. hodginsii* under different scenarios. We made two hypotheses, including: (1) climatic variables are the dominant drivers of *F. hodginsii* distribution, with soil properties and human influence playing secondary roles; and (2) climate change, especially under high‐emission scenarios, will result in significant habitat decline for *F. hodginsii*, regardless of human footprint intensity.

## 2 Materials and Methods

### 2.1 Species Occurrence Data and Study Area

The Wuyi Mountains, situated in southeastern China, have altitudes ranging from 200 m to 2158 m and are a region of exceptional ecological significance, serving as a refuge for several rare and endemic plant species. As a key species in the Wuyi Mountains, *F*. *hodginsii* plays an important role in the local forest ecosystem and is sensitive to climate variation. These mountains also host the largest retained primary mid‐subtropical, subtropical forests, which are also considered exceptional ecological and tourism resources (Chen 1999). The unique ecosystem, with its attractions to tourists and vulnerability to climate change, requires an understanding of distribution dynamics under climate change and human activities for species conservation. The Wuyi Mountains can be understood in both a narrow and a broad sense. In the narrow sense, it refers specifically to the Wuyi Mountain National Park. In the broader sense, the Wuyi Mountains extend beyond the boundaries of the national park, spanning a larger area across the border between Fujian and Jiangxi Provinces. We focus our study on the broader sense of the Wuyi Mountains, given that these areas represent a more comprehensive ecological landscape, providing a wider range of habitats and environmental gradients that are crucial for understanding the distribution and dynamics of *F. hodginsii*.

**FIGURE 1:**
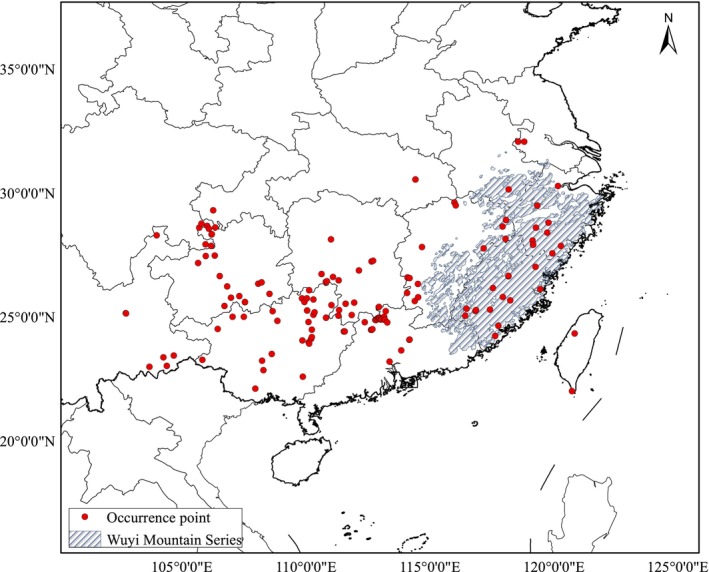
Distribution of occurrence point for *Fokienia hodginsii* retrieved from Global Biodiversity Information Database (GBIF, https://www.gbif.org/, accessed on May 10th 2024) and Chinese Virtual Herbarium (CVH, http://www.cvh.ac.cn/) (red points) and area of broad sense the Wuyi Mountain derived from Global Mountain Biodiversity Assessment (GMBA) Mountain Inventory v2 (Snethlage et al. 2022a, 2022b).

The species occurrence for *F. hodginsii* was collected from the Global Biodiversity Information Database (GBIF, https://www.gbif.org/, accessed on May 10th 2024) (Štípková et al. 2024) and Chinese Virtual Herbarium (CVH, http://www.cvh.ac.cn/) range‐wide. We retained 143 occurrence points in China by excluding repeated records using a 1 km grid as the filter (Figure 1). Given the various definitions of mountains and consequentially different mountain areas, we applied the definition and the vector map of Wuyi Mountains derived from Global Mountain Biodiversity Assessment (GMBA) Mountain Inventory v2 (standard) (Snethlage et al. 2022a, 2022b) (Figure 1).

### 2.2 Environmental Data

To study the *F. hodginsii* distribution under current climate conditions, we downloaded climate data in the normal period 1970–2000 and 2090s (averaged across 2081–2100) at the spatial resolution of 30 arcseconds (around 1 km grid) from WorldClim version 2.1 (https://worldclim.org/data/index.html, accessed on May 10th 2024) (Fick and Hijmans 2017). To project *F. hodginsii* distribution under future climate scenarios, we generated ensemble models using 13 available Generalized Circulation Models (GCMs) in the Coupled Model Intercomparison Project Phase 6 (CMIP6) from the Intergovernmental Panel of Climate Change (IPCC) 6th report, under the shared socioeconomic pathways SSP126 and SSP585. SSP126 represents a sustainable development pathway with low greenhouse gas emissions, while SSP585 represents a high emission pathway with minimal climate policies (Eyring et al. 2016). We retained 19 climatic variables from WorldClim version 2.1 for the following analysis: (Table 1).

**TABLE 1:** Climate variables used in this study.

Abbreviations	Description	Unit
**bio1**	**Annual average temperature**	**°C**
**bio2**	**Mean diurnal range (mean of montly (max temp ‐ min temp))**	**°C**
bio3	Isothermality (Bio2/Bio7) (*100%)	%
bio4	Temperature seasonality (standard deviation)	%
bio5	Max temperature of warmest month	°C
bio6	Min temperature of coldest month	°C
**bio7**	**Temperature annual range (MWMT‐MCMT)**	**°C**
bio8	Mean temperature of wettest quarter	°C
bio9	Mean temperature of driest quarter	°C
bio10	Mean temperature of warmest quarter	°C
bio11	Mean temperature of coldest quarter	°C
**bio12**	**Annual Precipitation**	**mm**
bio13	Precipitation of wettest month	mm
**bio14**	**Precipitation of driest month**	**mm**
**bio15**	**Precipitation seasonality (standard deviation)**	**mm**
bio16	Precipitation of wettest quarter	mm
bio17	Precipitation of driest quarter	mm
**bio18**	**Precipitation of warmest quarter**	**mm**
bio19	Precipitation of coldest quarter	mm

*Note:* Selected climate variables are in bold (see Section 2.3).

We downloaded 15 continuous topsoil variables (Table 2) from the Harmonized World Soil Database (HWSD, http://www.fao.org/land‐water/databases‐and‐software/hwsd/en/, accessed on May 10th 2024) at a spatial resolution of 30 arcseconds. HWSD was considered one of the most comprehensive soil databases.

**TABLE 2:** Soil variables used in this study.

Abbreviations	Description	Unit
T_GRAVEL	Topsoil gravel content	%vol.
**T_SAND**	**Topsoil sand fraction**	**%wt.**
T_SILT	Topsoil silt fraction	%wt.
T_CLAY	Topsoil clay fraction	%wt.
T_USDA_TEX_CLASS	Topsoil USDA texture classification	name
T_REF_BULK_DENSITY	Topsoil reference bulk density	kg/dm^3^
T_BULK_DENSITY	Topsoil bulk density	kg/dm^3^
T_OC	Topsoil organic carbon	%wt.
**T_PH_H2O**	**Topsoil pH (H_2_O)**	**−log (H+)**
T_CEC_CLAY	Topsoil cation exchange capacity (clay)	cmol/kg
T_CEC_SOIL	Topsoil caption exchange capacity (soil)	cmol/kg
T_TEB	Topsoil TEB	cmol/kg
T_CACO3	Topsoil calcium carbonate	%
T_CACO4	Topsoil gypsum	cmol/kg
T_ESP	Topsoil sodicity percentage	%
T_ECE	Topsoil salinity	dS/m

*Note:* Selected climate variables are in bold (see Section 2.3).

To reflect the impacts of human activities, we downloaded the human footprint index at a spatial resolution of 30 arcseconds from the Center for International Earth Science Information Network (CIESIN, https://sedac.ciesin.columbia.edu/data/set/wildareas‐v3‐2009‐human‐footprint). The human footprint index is measured using eight variables, including built‐up environments, population density, electric power infrastructure, croplands, pasture lands, roads, railways, and navigable waterways (Venter et al. 2016, 2018).

### 2.3 Model Building and Selecting

We built the models using presence data as the dependent variable and climate, soil, and human activities for 1970–2000 as independent variables. To simplify the analysis, following the idea of Koeling (2000), we first built a correlation matrix with all environmental variables for each occurrence point and excluded one variable from each pair of variables that were highly correlated to each other (absolute value of Pearson correlation coefficient > 0.85). We selected directly measured variables and annual variables over the derived variables and monthly variables. Permutation importance was used to evaluate the predictive contribution of each environmental variable. We further discarded variables with little contribution to the testing model until we retained 10 environmental variables for the development of the final models.

These selected environmental variables were used as predictors to calibrate four widely applied species distribution models (SDMs): the Maximum Entropy model (MaxEnt), random forest (RF), generalized linear models (GLM), and boosted regression trees (BRT). Before modeling, we randomly selected 1500 points as the pseudo‐absence background points. For each model, we used a 5‐fold cross‐validation. In each of five replicates, we selected 75% of the data points as the training set and 25% of the data points as the validation set. Model performance was evaluated based on three widely accepted metrics: the Area Under the Receiver Operating Characteristic Curve (AUC), the True Skill Statistic (TSS), and the Kappa coefficient. The mean values of these metrics across the five replicates were calculated for each model. The best‐performing model was identified by jointly considering all three indices to ensure both discriminative power, accuracy, and agreement consistency.

### 2.4 Suitable Area Classification

To classify the habitat suitability under different climate scenarios, we defined the area with a distribution probability of 0 to 0.1 as unsuitable, 0.1 to 0.3 as low suitability, 0.3 to 0.5 as median suitability, and 0.5 to 1 as high suitability (He et al. 2019; Sun, Qiu, et al. 2020). The area with high suitability was defined as the presence of *F. hodginsii*. To investigate the impacts of human activities on species distributions, we divided the human footprint index into low intensity (≤ 8) and high intensity (> 8) using their median in the Wuyi Mountains as the cutoff. Given that there is no available human footprint index prediction in the future, we applied the current human footprint index under SSP126 and SSP585 to investigate the effects of human activities. The corresponding distribution area change under different climate scenarios and human activity levels was calculated and categorized into three categories: stability (overlapping distribution areas in 1970–2000 and 2090s), loss (presence in 1970–2000 but absence in 2090s), and expansion (absence in 1970–2000 but presence in 2090s). Data analysis was conducted using packages “dismo,” “rJava,” “randomForest,” “caret,” “pROC” in R version 4.1.2 (R Core Team 2018). Visualization was conducted using package “ggplot2” in R version 4.1.2 and ArcGIS version 10.8.

### 2.5 Prediction Uncertainty Estimation

To assess the reliability of model predictions in extrapolated environments, we applied the Dissimilarity Index (DI) and Area of Applicability (AOA) framework, following the method of Meyer and Pebesma (2021). The DI measures the multivariate environmental distance between a prediction location and the training data in a standardized and importance‐weighted predictor space. All environmental predictor variables were standardized using the mean and standard deviation derived from the training dataset and were weighted by their normalized importance values extracted from the fitted SDM model (MaxEnt was selected as the final SDM as described in Section 3.1; therefore, permutation importance was used as weight).

For each prediction location X, the DI was calculated as the minimum Euclidean distance to all training points in the weighted standardized predictor space and then normalized by the average pairwise Euclidean distance among training points (Equations 1 and 2). All variables were standardized and weighted by normalized permutation importance before Euclidean distance was calculated.

(1) $$d\left(X\right)=\min_{X_{i}\in \tau }\left\{{\left\|W\left(X-X_{i}\right)\right\|}_{2}\right\}$$

(2) $$\mathrm{DI}\left(X\right)=d\left(X\right)/\bar{d\left(X_{l}\right)}$$

where $d\left(X\right)$ is the multivariate Euclidean distance calculation for a location X, the multivariate distance was calculated using retained 10 standardized environmental variables; τ denotes the set of training samples; W is a diagonal matrix of normalized permutation importance weights obtained from the selected SDM (MaxEnt); ${\left\|\cdot \right\|}_{2}$ is the Euclidean norm; $\mathrm{DI}\left(X\right)$ is the Dissimilarity Index for a location X; $\bar{d\left(X_{l}\right)}$ is the average pairwise Euclidean distance among training points, which is calculated using the same retained 10 standardized environmental variables weighted by the normalized permutation importance.

AOA was defined as the region where DI values fell within the range of nonoutlier DI values derived from the training set. Outliers were identified using the Tukey rule (i.e., DI values exceeding $Q_{3}+1.5\times \mathrm{IQR}$, where $Q_{3}$ is the 75th percentile and IQR is the interquartile range between $Q_{3}$ and $Q_{1}$). DI values were obtained through a 5‐fold cross‐validation procedure on the training data to ensure generalizable and conservative thresholding. However, the Tukey‐based threshold for AOA may be too conservative, and threshold determination varies across applications. To better characterize extrapolation‐related uncertainty, particularly since MaxEnt does not provide direct measures of prediction accuracy, we further examined the relationship between DI and the standard deviation (SD) of predictions across model replicates.

We followed the ODMAP (version 1.0) protocol (Zurell et al. 2020) and provided the full checklist in Table S1.

## 3 Results

### 3.1 Model Performance Assessment

**FIGURE 2:**
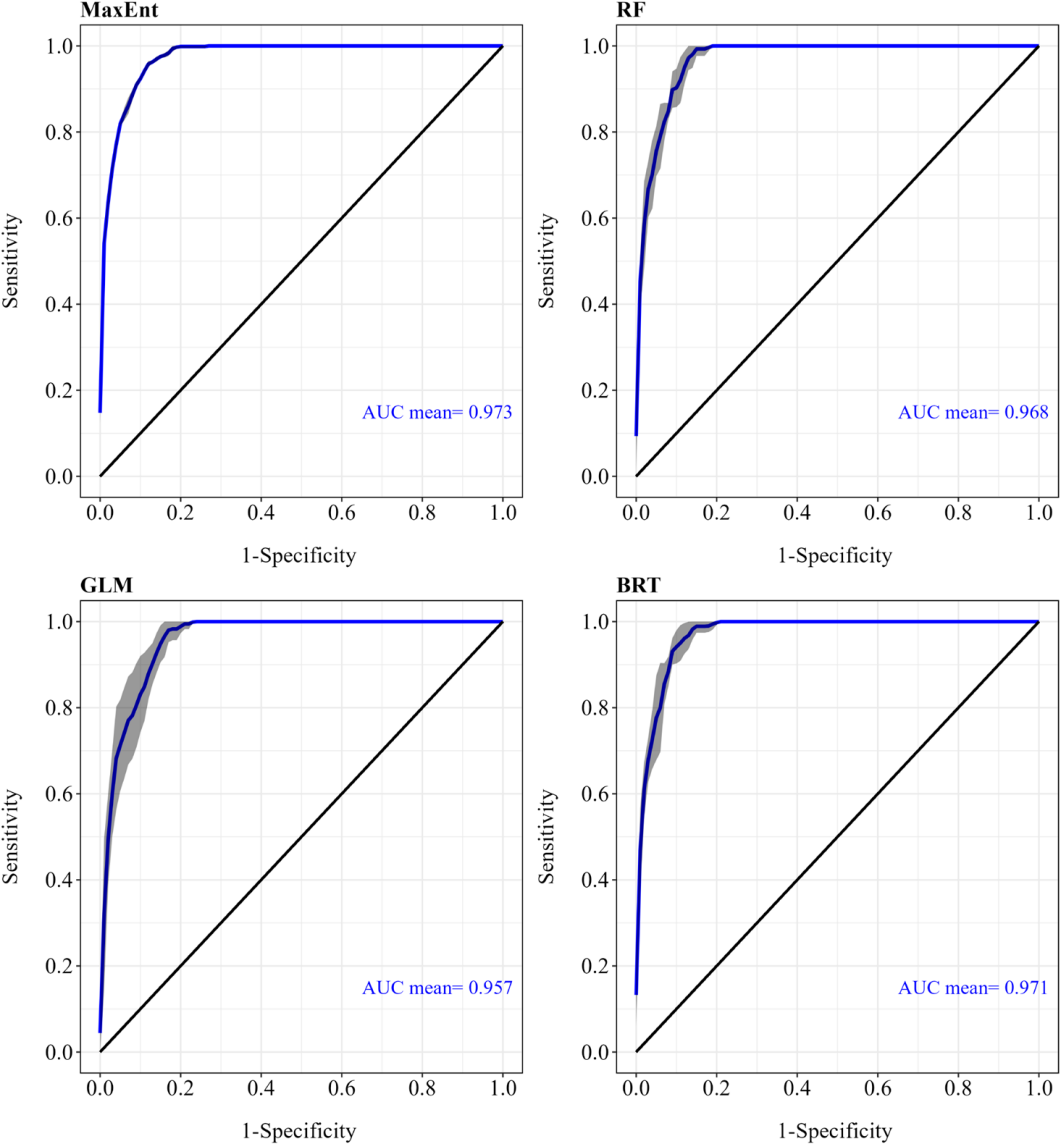
Receiver operating characteristic (ROC) curves for predicting species distribution of *Fokienia hodginsii* using the Maximum Entropy model (MaxEnt), random forest (RF), generalized linear models (GLM), and boosted regression trees models (BRT); sensitivity and specificity are assessed on test datasets over 5 times repeated runs. The gray band surrounding each ROC curve represents the ± standard deviation derived from the resampling replicates.

The ROC curves demonstrate high predictive performance across all models, with the MaxEnt model achieving the highest mean AUC value of 0.973, indicating superior discriminatory ability compared to the RF (AUC = 0.968), GLM (AUC = 0.957), and BRT (AUC = 0.971). MaxEnt also obtained the highest TSS (0.704), suggesting good classification accuracy. However, MaxEnt had a lower Kappa (0.395) compared to other models. RF had a moderate TSS (0.602) and a height Kappa (0.653). GLM showed the lowest TSS (0.437) among the four models and a moderate Kappa (0.546), despite a reasonably high AUC (0.957). BRT had a moderate TSS (0.576) and a moderate Kappa (0.633). Overall, MaxEnt exhibited the strongest discriminatory power and reasonable prediction consistency and therefore was chosen as the final SDM in the subsequent analysis (Figure 2 and Table 3). According to the permutation importance results of the MaxEnt model (Table 4), temperature annual range (bio7) was identified as the most influential environmental predictor, with a permutation importance of 47.6%. This was followed by the mean diurnal range (of temperature) (bio2, 17.3%) and precipitation of the driest quarter (bio18, 14.8%), indicating that both climatic variability and seasonal extremes play critical roles in shaping the distribution of *F. hodginsii*. Human footprint index also played an important role in affecting species distribution with a permutation importance of 8.9%, suggesting nonnegligible effects from human activities. In contrast, soil variables, including topsoil sand fraction (0.2%) and topsoil pH, had the lowest influence, suggesting that climate variables are the primary drivers of the species' potential habitat suitability (Table 3).

**TABLE 3:** Predictive performance of four species‐distribution modeling algorithms. Values of the area under the ROC curve (AUC), true skill statistic (TSS) and Cohen's kappa are the means from five independent repeated runs of each model; larger values indicate stronger discriminatory power and classification agreement.

Model	AUC	TSS	Kappa
MaxEnt	0.973	0.704	0.395
Random forest	0.968	0.602	0.653
Generalized linear model	0.957	0.437	0.546
Boosted regression tree	0.971	0.576	0.633

**TABLE 4:** Permutation importance of retained environmental variables used in building MaxEnt model for *Fokienia hodginsii*.

Abbreviations	Description	Permutation importance (%)
bio7	Temperature annual range	47.6
bio2	Mean diurnal range	17.3
bio14	Precipitation of driest month	14.8
HF	Human footprint index	8.9
bio1	Annual mean temperature	6.1
bio18	Precipitation of warmest quarter	2.4
bio12	Annual precipitation	1.8
bio15	Precipitation seasonality	0.9
t_sand	Topsoil sand fraction	0.2
t_ph_h2o	Topsoil pH (H_2_O)	0.1

**FIGURE 3:**
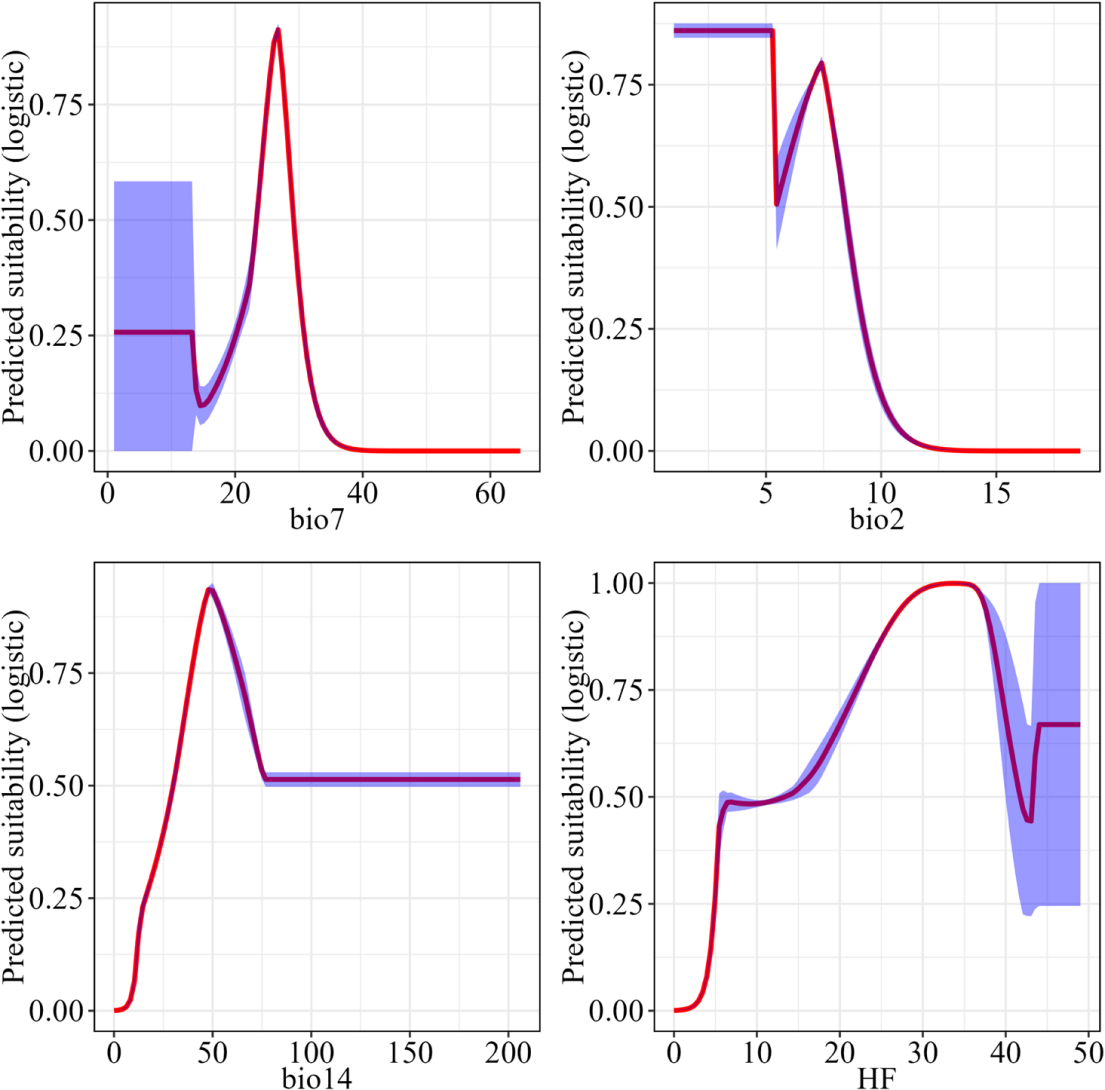
Univariate response curves of four most important climate variables, including bio7 (temperature annual range), bio2 (mean diurnal range), bio14 (precipitation of driest month), and human footprint index (HF), in predicting species distribution (Logistic output) of *Fokienia hodginsii* using MaxEnt model.

For temperature variables, the response curve for bio7 (temperature annual range) displayed a distinct peak around 25°C, suggesting a preference for environments with some but not extreme temperature variability; bio2 (mean diurnal range) showed the highest suitability at approximately 0–7.5 C, with a marked drop beyond 8 C, suggesting a preference for moderate daily temperature fluctuations; bio14 (precipitation of driest quarter) showed a peak in around 50 mm, with a plateauing beyond approximately 50 mm. The HF (human footprint) showed a steady increase in improving suitability, peaking near 30–35, but dropped markedly above 40, suggesting that *F. hodginsii* may tolerate or even benefit from moderate anthropogenic disturbances, possibly due to habitat mosaics created in human‐modified landscapes (Figure 3).

### 3.2 Suitable Habitat Distribution

**FIGURE 4:**
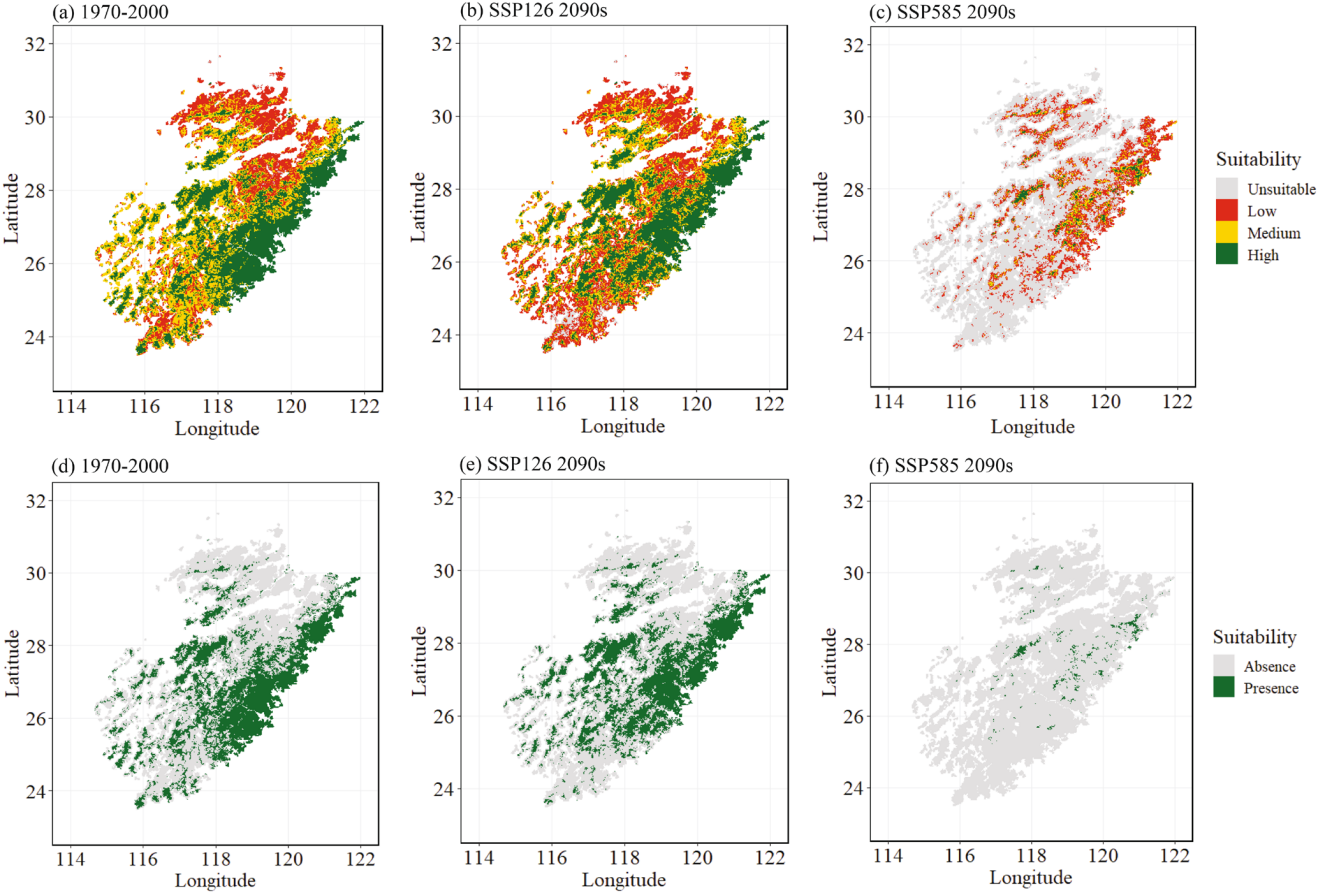
Predicted habitat suitability levels of *Fokienia hodginsii* (unsuitable, probability < 0.1; low suitability, 0.1–0.3; median suitability, 0.3–0.5; and high suitability, > 0.5) in 1970–2000 (a) and 2090s (2080–2100) under shared socioeconomic pathways (SSPs)126 (b) and 585 (c) across area of the Wuyi Mountains; predicted presence area distribution (median and high suitability) in 1970–2000 (d) and 2090s under SSPs126 (e) and 585 (f) across area of the Wuyi Mountains.

In the normal period of 1970–2000, the suitable habitat for *F. hodginsii* was estimated to spread from the central to southeastern regions of the Wuyi Mountains, encompassing a total area of 96458.9 km^2^. The areas of low suitability were mainly distributed in the northern Wuyi Mountains, with some scattered patches in the south, covering 60574.8 km^2^ (Figure 4a,d; Figure 6a).

**FIGURE 5:**
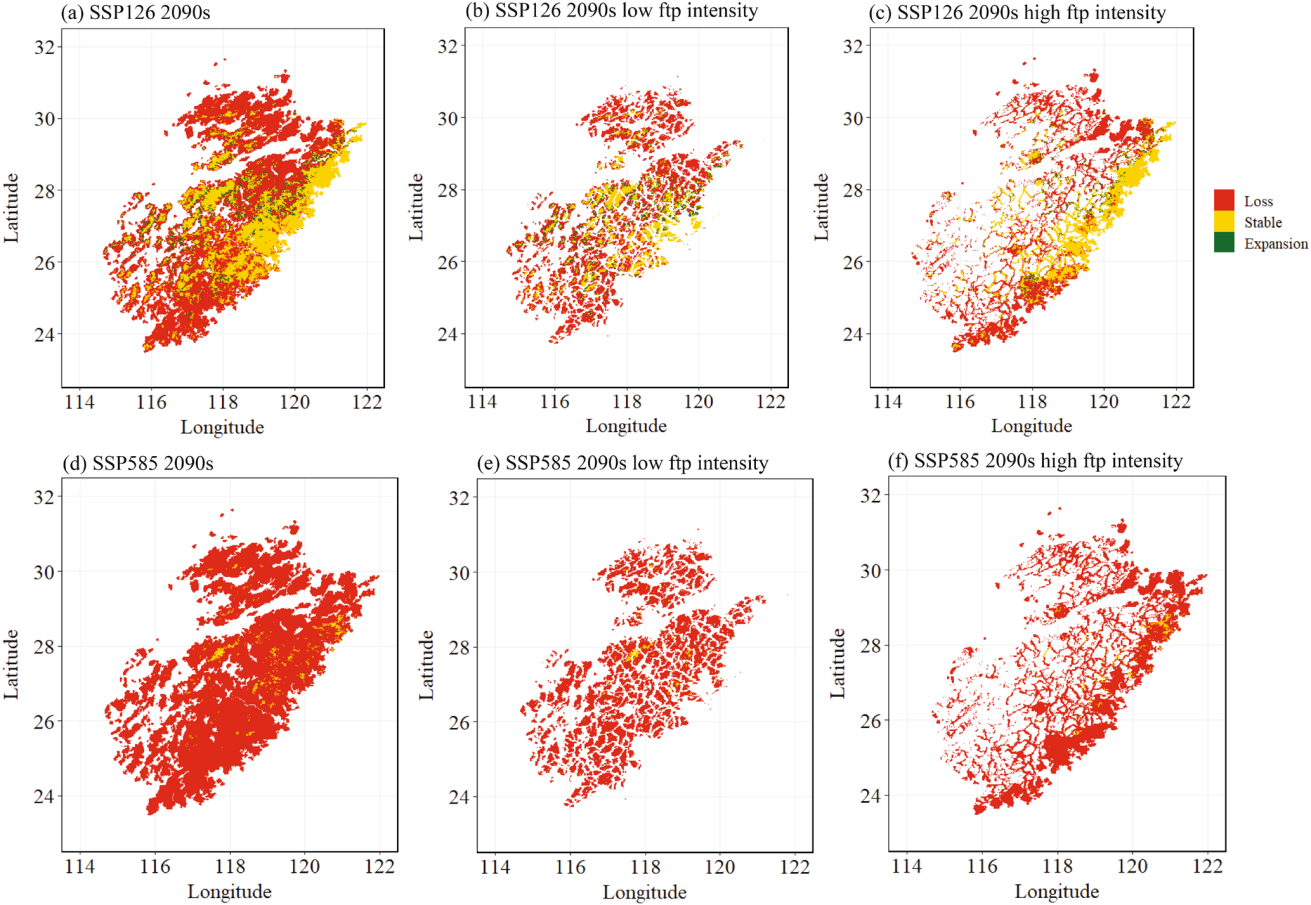
Predicted species distribution area change across the area of *Fokienia hodginsii* in the Wuyi Mountains under shared socioeconomic pathway (SSP)126 (a) and SSP585 (d); predicted species distribution area changes with low human footprint (ftp) intensity under SSP126 (c) and SSP585 (c); predicted species distribution area changes with high human footprint (ftp) intensity under SSP126 (e) and SSP585 (f).

**FIGURE 6:**
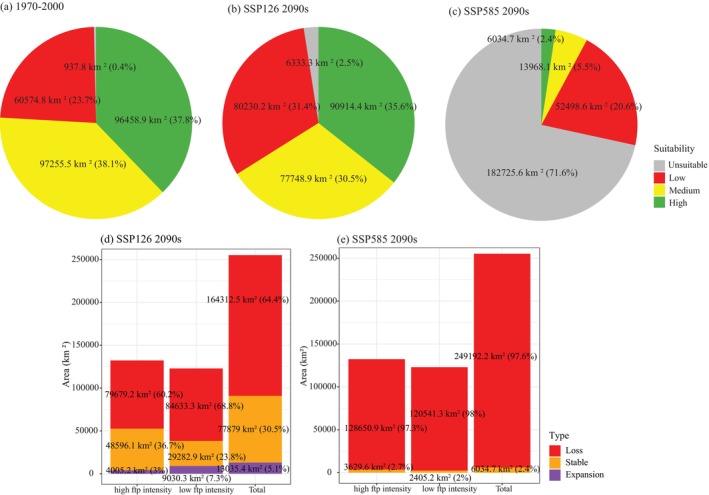
Percentage of predicted habitat suitability levels of *Fokienia hodginsii* for (unsuitable, probability < 0.1; low suitability, 0.1–0.3; median suitability, 0.3–0.5; and high suitability, > 0.5) in 1970–2000 (a) and 2090s (2080–2100) under shared socioeconomic pathways (SSPs)126 (b) and 585 (c); percentage of predicted species distribution area change in 2090s under SSPs126 (d) and 585 (e) for high footprint (ftp) intensity and low ftp intensity.

Under both SSP126 and SSP585 scenarios, the future (2090s) suitable habitat area for *F. hodginsii* was projected to experience a general decline. Under the SSP126 scenario, highly suitable habitats were slightly reduced to 90914.4 km^2^, while areas with median, low suitability, or even unsuitability were expected to expand. This trend is evident in the spatial distribution of suitability levels (Figure 4b,e), and the proportional change in habitat categories (Figure 5a; Figure 6b,d). In contrast, the SSP585 scenario showed a more pronounced decline, with many areas currently classified as highly suitable shifting to low suitability, and moderately to poorly suitable areas transitioning to unsuitable (Figure 4c,f; Figure 5c; Figure 6c,e). Overall, future habitat loss was expected to be more severe in the southern and northern regions of the Wuyi Mountains. By the 2090s, the suitable habitat was projected to decrease to 6034.7 km^2^ under the SSP585 scenario (Figure 6c).

By the 2090s, climate‐driven habitat degradation for *F. hodginsii* was projected to far exceed the impact of human disturbance, particularly under high‐emission scenarios. Both SSP126 and SSP585 projected predominantly habitat loss for *F. hodginsii* habitat regardless of human‐footprint intensity. Under SSP126, a pocket of stable suitability was predicted to persist in the more heavily populated eastern Wuyi Mountains (Figure 5c), accounting for 36.7% of the area of high human influence (Figure 6d). 23.8% of the area of low human influence was predicted to persist (Figure 6d) and distribute in scattered patches (Figure 5b). By contrast, a more severe decline of habitat is expected under the SSP585 scenario regardless of human footprint level (Figure 5e,f), and over 90% habitat loss is predicted in total (Figure 6e).

### 3.3 Prediction Uncertainty Estimation

**FIGURE 7:**
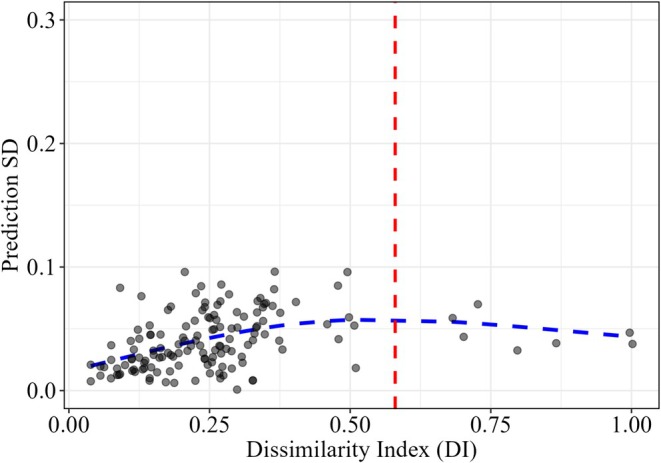
Relationship between prediction SD (standard deviation) and Dissimilarity Index (DI) derived from the MaxEnt model built on 143 occurrence points for *Fokienia hodginsii* with five‐fold cross‐validation; red vertical dashed line is the Area of Applicability (AOA) defined by the Tukey rule.

**FIGURE 8:**
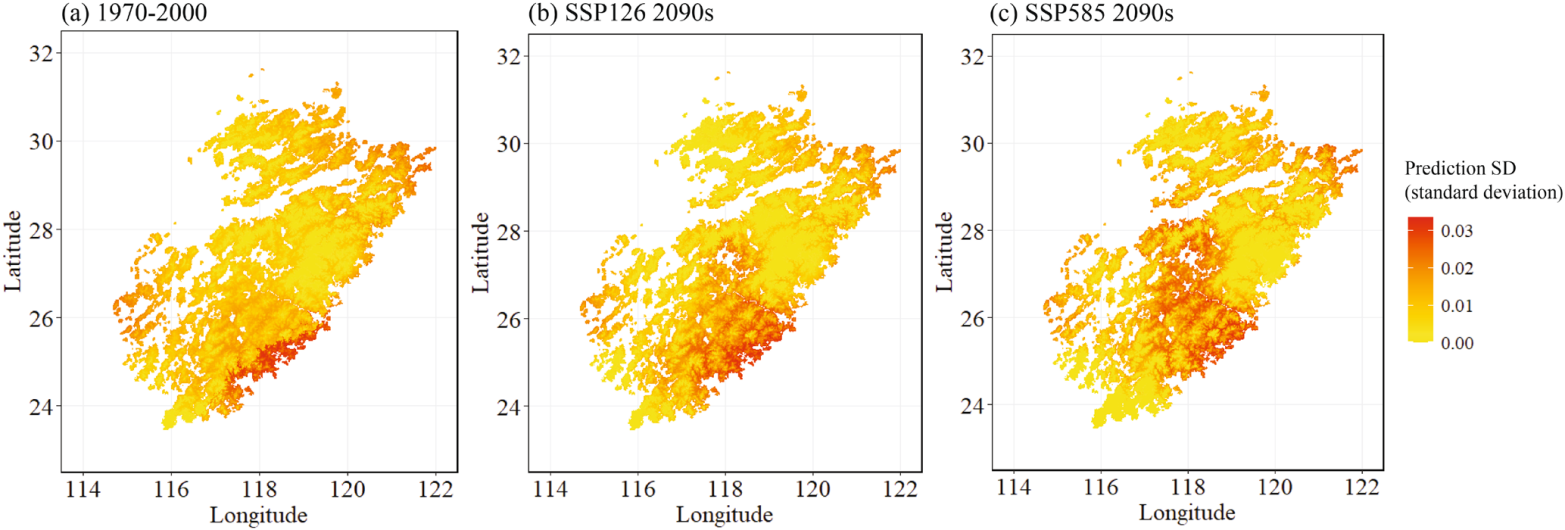
Prediction standard deviation (SD) associated with dissimilarity index (DI) for MaxEnt model in 1970‐2000 (a); in 2090s (2080‐2100) under shared socioecnomic pathway (SSP)126 (b); and in 2090s under SSP585 (c) across the Wuyi Mountains areas.

Predictive uncertainty of MaxEnt model remained low and relatively stable across most of the DI range, suggested by the no clear monotonic increase in SD as DI increased (Figure 7). The red dashed line indicates the AOA threshold based on the Tukey rule. Most training samples fall within this boundary, with a small number of points exhibiting higher DI values beyond the AOA threshold. However, these high‐DI points did not correspond to markedly elevated prediction SD, implying that model uncertainty remained moderate even near the extrapolation limit. Using a generalized additive model (GAM), the prediction SD was estimated and visualized across Wuyi Mountains. The highest predicted SD was approximately 0.03 for the logistic output (habitat suitability) of the MaxEnt model, under both the baseline period (1970–2000) and future scenarios in the 2090s under SSP126 and SSP585 (Figure 8).

## 4 Discussion

### 4.1 Effects of Environmental Factors on the Distribution of *F. Hodginsii*

Using SDMs to assess the habitat suitability, we found that climate variables had the highest permutation importance in deciding the distribution of *F. hodginsii*. Given that occurrence records of *F. hodginsii* span across southern China and *F. hodginsii* can tolerate a range of soil conditions, with a general preference for acidic sandy loams (Thomas and Luu 2022), climate emerged as the dominant factor in predicting the species' occurrence probability and identifying suitable habitats for *F. hodginsii*. This is consistent with ecological niche theory, which highlights climate as a primary driver of species' spatial distributions (Davis and Shaw 2001; Woodward and Williams 1987). Temperature is typically considered the most important climate driver for growth, phenological events, and metabolic activities for conifer tree species (Guo et al. 2020; Li et al. 2012).

Low winter temperature is considered limiting survival and growth of conifers due to their potential for frost damage (Pearce 2001). Although subtropical areas have warm temperature, making frost damage less likely to occur to local conifer trees, it does not exclude the possibility of potential frost damage, given that conifers in subtropical ecosystems are rarely experienced selection for frost tolerance. Lack of frost tolerance adaptation as their counterpart in northern areas may lead to high mortality of *F. hodginsii* under some historical extreme climate events, such as the freezing weather in southern China in 2008 (Zhang et al. 2011). In addition, *F. hodginsii* in the Wuyi Mountains distributes across a relatively large elevation range, and frost is not uncommon in the winter of subtropical mountains. Our results indicated that the temperature annual range had the highest permutation importance, which could be related to potential winter frost in Wuyi Mountains.

Plant growth, such as cell division and elongation, is dependent on temperature and is even mainly driven by temperature. The increasing temperature may lead to a longer growing season (Keenan et al. 2014) and faster development of spring photosynthetic capacity (Linkosalo et al. 2017) that benefits tree growth. However, the conifer trees exposed to full sunlight in high temperature are recorded with significant physiological and biochemical disorders, leading to defoliated canopy, reduction in photosynthesis rate, and weakened defense mechanisms (Tiwari et al. 2021; Kunert et al. 2022). Rising temperatures may exacerbate drought stress under limited water availability and are recorded with consequent tree growth decline in subtropical regions in southern China (Jing et al. 2022; Su et al. 2021).

While soil properties play a secondary role, their interaction with climate factors, such as soil texture and composition, substantially affects nutrient availability, water retention under increased drought stress, and can further shape habitat suitability (Binkley and Fisher 2019). In general, topsoil silt, clay fraction, and bulk density reflect the proportion of soil particles at different sizes, which is related to the soil's water retention capacity, aeration, pH, organic carbon content, and soil's cation exchange capacity (Khaledian et al. 2017; Razzaghi et al. 2020; Rodella et al. 1995; Sun, Lee, et al. 2020). These soil features are critical for root development and overall plant health and growth. Our results indicate that *F. hodginsii* prefers moderately acidic and sandy soil, which aligns with previous studies (Thomas and Luu 2022). This highlights the importance of balanced nutrient availability and soil acidity, which affect nutrient availability and microbial activity, ultimately influencing habitat suitability. Nevertheless, soil variables exert a comparatively smaller influence on the distribution of *F. hodginsii* than do climatic factors.

Although human activities did not dominate the distribution of *F. hodginsii*, we retained the human footprint index given that it might not be fully reflected by climate and soil variables, and it has ecological implications as suggested by other studies (Fuller et al. 1998; Sun, Qiu, et al. 2020). The response to the human footprint indicates that *F. hodginsii* may be able to tolerate human disturbances or even benefit from moderate human activities. In regions with moderate to high human activity, more favorable growth conditions could be provided for tree species by artificial facilitating introduction or planting. Human‐modified environments resulting from forestry activities can also create suitable microhabitats or reduce competition in these areas for specific tree species. These characteristics may contribute to the increasing habitat with higher human activities. However, our study was unable to identify specific types of human activities that benefit the distribution of *F. hodginsii*. Regardless of the type of human activities, our predictions indicate a trend of habitat loss under climate change, specifically under the high emission scenario. This trend is projected to occur at both low and high levels of human influence, underscoring the need for conservation‐oriented human interventions.

### 4.2 Model Prediction Uncertainty Assessment

To assess model uncertainty for the selected MaxEnt, we applied the DI and AOA. Although the fitted GAM did not show a sharp increase in prediction standard deviation with increasing DI beyond AOA, the limited number of occurrence points and the potential for extrapolation highlight the need for caution. Unlike RF and BRT, MaxEnt does not allow direct prediction accuracy assessment. Instead, we evaluated uncertainty using the SD across replicate models. Despite these limitations, the overall spatial variation in predictions across the Wuyi Mountains was basically consistent for the MaxEnt model.

### 4.3 Changing Geographical Distribution of *F. Hodginsii* and Its Potential Influences

The response curves in our study highlight the species' preference for certain ranges of temperature and precipitation levels, and soil conditions. This phenomenon of local adaptation is extensively documented for forest tree species, as species tend to retain their niche and related ancestral traits (Atkins and Travis 2010; Bocedi et al. 2013; McKay et al. 2005; Wiens and Graham 2005). Phylogeographic evidence further supports this adaptive conservatism in *F. hodginsii*, which has undergone a long evolutionary history shaped by paleoclimatic oscillations since the Neogene. Genetic analyses reveal two major lineages that diverged in the early Miocene, likely driven by the onset of the Asian monsoon, habitat fragmentation during the last glacial period and local expansion during postglacial periods (Yin et al. 2021). Such evolutionary divergence and local adaptation have constrained the species' range adjustments to environmental change. However, under rapid contemporary climate warming, the natural migration and selection rates of *F. hodginsii* may not keep pace. As our results indicate, *F. hodginsii* presents a trend of habitat shrinking, leaving the original habitat potentially to be replaced by other plant species. Specifically, under the most severe global warming scenario, the maladaptation is predicted to be exacerbated.

The reduction in suitable habitats could negatively impact the regional forest economy, given the ornamental and medicinal value of *F. hodginsii*. For example, Huang et al. (2013) reported that the medicinal applications of *F. hodginsii* contribute significantly to local livelihoods, underscoring the socio‐economic ramifications of habitat loss. Recent phytochemical analyses have further highlighted the economic potential of *F. hodginsii*, the essential oils extracted from its leaves were found to be rich in α‐pinene, limonene, and myrcene, exhibiting strong antimicrobial activity against *Enterococcus faecalis* and *Bacillus cereus* (Thanh et al. 2023). The loss of habitat can also diminish the ecosystem services it provides, such as carbon sequestration, soil stabilization, and water regulation, thereby affecting the broader environmental health and resilience of the region. Given that the Wuyi Mountains host the largest retained primary mid‐subtropical forests (Chen 1999), the changing distribution and abundance of *F. hodginsii* can disrupt the structure of existing plant communities and the ecological relationships within them, potentially leading to unpredictable ecological risks. Tourism in the Wuyi Mountains may also suffer as *F. hodginsii* plays an important role in the natural beauty with local characteristics and biodiversity that attract visitors. Forests losing this species could become less appealing; the replacement of *F. hodginsii* by less familiar or less iconic species might reduce the unique character of these forests, impacting cultural and recreational experiences for tourists, therefore leading to a decline in ecotourism revenue.

### 4.4 Implications and Limitations

The potential risk of maladaptation and decreasing suitable habitat emphasizes the critical need for incorporating climate projections into conservation planning and management strategies to mitigate potential adverse impacts on forest ecosystems. Relevant in situ and ex situ conservation activities, such as assisted migration, tree improvement, and seed banks, should be considered and conducted. For the narrow sense of the Wuyi Mountains (national park), conservation practices should focus on the buffer zones and transition areas surrounding the core zones.

In our study, we assumed constant soil conditions and human activities for future predictions, which could oversimplify their dynamic interactions with climate factors. Incorporating temporal changes in soil properties, such as organic carbon fluxes, and high‐resolution human activity data could enhance model accuracy in future studies. Additionally, we did not delve into the economic value of *F. hodginsii*. Given the significance of the Wuyi Mountains for tourism, the impacts of shifting distributions of *F. hodginsii* should be further understood. This understanding is crucial for developing comprehensive conservation strategies that not only protect the species but also sustain the economic benefits derived from forestry and tourism in the region.

## 5 Conclusions

The rapid climate change poses a great risk to the survival and distribution of *F. hodginsii* in the Wuyi Mountains areas. We built multiple species distribution models and selected the MaxEnt model to predict the habitat suitability of *F. hodginsii* using variables of climate, soil, and human footprint index under two climate change scenarios, SSP126 and SSP585. The final model showed good discriminative power and moderately high prediction accuracy, with climate variables, specifically temperature variables, dominantly affecting the distribution of *F. hodginsii*. The results indicated a trend of declining species range, specifically under the high emission climate change scenario. Overall, the predicted massive habitat loss highlights the necessity of extra attention for conservation practices; therefore, to mitigate the adverse effects of climate change on *F. hodginsii* in the Wuyi Mountains region.

## Author Contributions

**Dawei Luo:** data curation (lead), formal analysis (equal), project administration (lead), software (lead), supervision (lead), writing – original draft (lead). **Tongli Wang:** writing – review and editing (equal). **Jiejie Sun:** formal analysis (lead), project administration (lead), supervision (lead), writing – review and editing (lead). **Xiali Guo:** writing – review and editing (equal). **Mingliang Peng:** writing – review and editing (equal). **Hongxi Shen:** writing – review and editing (equal). **Jing Qian:** writing – review and editing (equal).

## Conflicts of Interest

The authors declare no conflicts of interest.

## Supporting information


**Data S1:** ece372887‐sup‐0001‐supinfo.docx.

## Data Availability

The occurrence and environment data for our modeling, R script, README, environmental data for prediction are available from the open data repository (link: https://figshare.com/s/244578793876b63da60f). The species occurrence data for *Fokienia hodginsii* originally from Global Biodiversity Information Database (GBIF, https://www.gbif.org/) and Chinese Virtual Herbarium (CVH, http://www.cvh.ac.cn/). The climate data is originally from WorldClim version 2.1 (https://worldclim.org/data/index.html). Soil data is originally from the Harmonized World Soil Database (HWSD, http://www.fao.org/land‐water/databases‐and‐software/hwsd/en/). Human footprint index is originally from the Center for International Earth Science Information Network (CIESIN, https://sedac.ciesin.columbia.edu/data/set/wildareas‐v3‐2009‐human‐footprint).
