# Supplementary material for: Conservation Challenges and Opportunities for Fokienia hodginsii in the Wuyi Mountains Under Climate Change and Human Influence
Source: Ecol Evol. 2026 Jan 7;16(1):e72887. doi: 10.1002/ece3.72887 (PMC12779470; doi:10.1002/ece3.72887)
Supplement: Supplementary file 1 — Data S1: ece372887‐sup‐0001‐supinfo.docx. [file ECE3-16-e72887-s001.docx]

**Supplementary Materials**

**Table S1** The five main ODMAP sections and list of ODMAP elements.

| **ODMAP section** | | | **ODMAP subsection** | | | **ODMAP elements** | | | | |
| --- | --- | --- | --- | --- | --- | --- | --- | --- | --- | --- |
|  | | **O**verview |  | | Authorship |  | | · Dawei Luo, Jiejie Sun, Xiali Guo, Mingliang Peng, Jing Qian | | |
|  |  |  |  |  |  |  |  | · Jiejie Sun | | |
|  |  |  |  |  |  |  |  | · To assess the current and future habitat suitability of *Fokienia hodginsii* under climate change and human influence using SDMs | | |
|  |  |  |  |  |  |  |  | · Pending assignment upon publication | | |
|  |  |  |  | | Model objective |  | | · SDM objective/purpose: | | |
|  |  |  |  |  |  |  |  | ecological inference / explanation | Mapping / interpolation | Forecast / transfer |
|  |  |  |  |  |  |  |  | · Main target output: To assess the current and future habitat suitability of *Fokienia hodginsii* under climate change and human influence using SDMs | | |
|  |  |  |  | | Taxon |  | | · *Fokienia hodginsii* | | |
|  |  |  |  | | Location |  | | · Wuyi Mountains, Southeast China | | |
|  |  |  |  | | Scale of analysis |  | | · Spatial Extent (Lon100 -125/ Lat15-35) | | |
|  |  |  |  |  |  |  |  | · Spatial resolution: 1km | | |
|  |  |  |  |  |  |  |  | · Time period: Baseline: 1970–2000; Projections: 2090s under SSP126 and SSP585 | | |
|  |  |  |  |  |  |  |  | · Temporal resolution: 30-year normal period average | | |
|  |  |  |  |  |  |  |  | · natural and political | | |
|  |  |  |  | | Biodiversity data overview |  | | · Observation type: field survey | | |
|  |  |  |  |  |  |  |  | · Response/data type: presence-only | | |
|  |  |  |  | | Type of predictors |  | | · Climate (bioclimatic), soil properties, human footprint index. | | |
|  |  |  |  | | Conceptual model |  | | · Hypotheses about species-environment relationships: Climate and anthropogenic factors will jointly determine the current and future distribution; loss expected under high-emissions scenarios | | |
|  |  |  |  | | Assumptions |  | | · Species distribution correlates with environmental suitability; niche conservatism; projections assume species equilibrium (cf. Table 2) | | |
|  |  |  |  | | SDM algorithms |  | | · MaxEnt, Random Forest, Boosted Regression Tree (BRT), Generalized Linear Model (GLM) | | |
|  |  |  |  |  |  |  |  | · Model complexity reduced by variable selection and algorithm-specific regularization | | |
|  |  |  |  |  |  |  |  | · Averaging/ensemble modelling used for climate | | |
|  |  |  |  | | Model workflow |  | | 1. Species occurrence records filtered (143 presences, 1-km grid thinning);  2. Environmental predictor layers collected (climate, soil, human footprint);  3. Correlation filtering and variable importance selection (final set of 10 predictors);  4. Four SDMs (MaxEnt, RF, BRT, GLM) calibrated and evaluated using 5-fold cross-validation;  5. Model with best performance (MaxEnt) selected for prediction;  6. Future suitability mapped under SSP126 and SSP585 using ensemble GCM outputs;  7. Uncertainty quantified using DI and SD analysis. | | |
|  |  |  |  | | Software, codes and data |  | | · R used for modeling (pakcages: e.g. dismo, randomForest, caret, pROC, rJava, ggplot2) | | |
|  |  |  |  |  |  |  |  | · Specify availability of codes: https://figshare.com/s/244578793876b63da60f | | |
|  |  |  |  |  |  |  |  | · Specify availability of data, https://figshare.com/s/244578793876b63da60f | | |
|  | | **D**ata |  | | Biodiversity data |  | | · *Fokienia hodginsii* (Dunn) A.Henry & H.H.Thomas | | |
|  |  |  |  |  |  |  | | · Details on taxonomic reference system:GBIF and CVH records | | |
|  |  |  |  |  |  |  | | · Ecological level (pop) | | |
|  |  |  |  |  |  |  | | · Biodiversity data source: GBIF (https://www.gbif.org/), CVH (http://www.cvh.ac.cn/); accessed May 10th, 2024 | | |
|  |  |  |  |  |  |  | | · Sampling design: Opportunistic occurrence records from aggregated databases; filtered with 1 km spatial grid to reduce clustering | | |
|  |  |  |  |  |  |  | | · Sample size per taxon: 143 unique occurrence records (after filtering duplicates) | | |
|  |  |  |  |  |  |  | | · Country/region masl: China; Wuyi Mountains region, 200–2,158 meters above sea level | | |
|  |  |  |  |  |  |  | | · Details on scaling: Spatial thinning at 1 km resolution; analysis scaled to 30 arc-second environmental data | | |
|  |  |  |  |  |  |  | | · Details on data cleaning/filtering steps, if applicable: Duplicate removal via 1 km grid; species identity checked against database taxonomy; no obvious outliers noted | | |
|  |  |  |  |  |  |  | | · Details on absence data collection, if applicable:NA | | |
|  |  |  |  |  |  |  | | · Details on background data derivation, if applicable: 1500 pseudo-absence/background points randomly sampled across study region; no bias correction used | | |
|  |  |  |  |  |  |  | | · Details on potential errors and biases in data, if applicable: e.g., misidentification potential | | |
|  |  |  |  |  | Data partitioning |  |  | · Selection of training data for model fitting:75% of presence points and pseudo-absences were randomly selected for training in each model run | | |
|  |  |  |  |  |  |  |  | · Selection of validation data: 25% of data used as validation set in each of five replicates for cross-validation; averaged results used for evaluation | | |
|  |  |  |  |  |  |  | | · Selection of test (truly independent) data:No independent test data used; internal 5-fold cross-validation only | | |
|  |  |  |  | | Predictor variables |  | | · State predictor variables used:climate variables, soil variables, human footprint index | | |
|  |  |  |  |  |  |  | | · Details on data sources: Climate: WorldClim v2.1 (1970–2000 baseline, 2090s projections), Soil: HWSD (FAO), Human Footprint: CIESIN (2009), all accessed May 10th, 2024 | | |
|  |  |  |  |  |  |  | | · Spatial resolution and spatial extent of raw data: 30 arcseconds (~1 km); extent matched Wuyi Mountains GMBA v2 boundaries | | |
|  |  |  |  |  |  |  | | · Map projection (coordinate reference system):WGS84 | | |
|  |  |  |  |  |  |  | | · Temporal resolution and temporal extent of raw data:30-year average | | |
|  |  |  |  |  |  |  | | · Details on data processing and on spatial, temporal and thematic scaling: e.g. upscaling/downscaling, transformations, normalisations, thematic aggregations (e.g. of land cover classes), measures to address spatial uncertaintie:All environmental predictors were processed to a uniform spatial resolution of ~1 km (30 arcseconds) to match the resolution of WorldClim v2.1. Climatic variables were derived from monthly data as long-term bioclimatic summaries. Soil and human footprint layers were resampled and aligned to the climate grid using bilinear interpolation (for continuous variables). No upscaling or downscaling was performed beyond this resolution. No thematic aggregation (e.g., land cover reclassification) was applied. To reduce spatial uncertainty, species occurrence points were spatially filtered using a 1 km thinning grid. Predictors were also standardized during model uncertainty estimation (DI and AOA analysis) using z-score normalization weighted by variable importance. | | |
|  |  |  |  |  |  |  | | · Details on measurements errors and bias: check GBIF and CVH | | |
|  |  |  |  |  |  |  | | · Details on dimension reduction of variable set, if applicable – if model-based, this should be contained in Model section (element: Details on pre-selection of variables):Dimension reduction was performed prior to modeling to address multicollinearity. We computed Pearson correlation coefficients among all candidate predictor variables and excluded one variable from each pair with correlation \|r\| > 0.85. Preference was given to ecologically interpretable and directly measured variables (e.g., bio1, bio7). Additionally, variables with low permutation importance in preliminary MaxEnt runs were discarded. The final model retained 10 predictors representing climatic, soil, and anthropogenic factors. | | |
|  |  |  |  | | Transfer data for projection |  | | · Details on data sources: Climate: WorldClim v2.1 (1970–2000 baseline, 2090s projections), Soil: HWSD (FAO), Human Footprint: CIESIN (2009), all accessed May 10th, 2024 | | |
|  |  |  |  |  |  |  | | · Spatial extent (Lon114 -122/ Lat22-32) | | |
|  |  |  |  |  |  |  |  | · Spatial resolution:1km | | |
|  |  |  |  |  |  |  |  | Time projections: 2090s under SSP126 and SSP585 | | |
|  |  |  |  |  |  |  |  | · temporal resolution:30-year average | | |
|  |  |  |  |  |  |  | | · Models and scenarios used:SSP126 and SSP585 | | |
|  |  |  |  |  |  |  | | · Details on data processing and scaling (see above) | | |
|  |  |  |  |  |  |  | | · Quantification of novel environmental conditions and novel environmental combinations: distance to training data | | |
|  | | **M**odel |  | | Variable pre-selection |  | | · Details on pre-selection of variables, see above | | |
|  |  |  |  | | Multicollinearity |  | | · Methods for identifying and dealing with multicollinearity (Dormann, et al. 2013) or justification if multicollinearity is not explicitly dealt with: correlation matrix is considered in variable selection | | |
|  |  |  |  | | Model settings |  | | · Models settings for all selected algorithms (including default settings of specific platforms/packages, weighting of data etc.) | | |
|  |  |  |  |  |  |  | | · Details on relevant model settings for extrapolation beyond sample range, if applicable: Default settings were used for all four algorithms unless otherwise stated. For MaxEnt, regularization multiplier was kept at the default (1.0), and background points were set at 1,500. Features were automatically selected by the software based on data properties. For Random Forest (RF), 500 trees were used with default settings in the randomForest R package. For Boosted Regression Tree (BRT), the gbm.step function was used with a tree complexity of 5, learning rate of 0.01, and a maximum of 5,000 trees. Generalized Linear Model (GLM) used a binomial logistic regression with stepwise variable selection. No additional weighting of presence/background data was applied. Each model was replicated five times using random partitions for cross-validation. | | |
|  |  |  |  | | Model estimates |  | | · Assessment of model coefficients: Model coefficients were explicitly assessed only for the Generalized Linear Model (GLM), where variable significance and direction of effect (positive/negative) were examined. For MaxEnt, Random Forest, and BRT, variable influence was assessed using permutation importance and response curves rather than traditional coefficients. | | |
|  |  |  |  |  |  |  | | · Details on quantification of uncertainty in model coefficients, e.g. model performance assessment were done using a 5-fold cross validaiton | | |
|  |  |  |  |  |  |  | | · Assessment of variable importance:Variable importance was assessed for each model using algorithm-specific metrics. For MaxEnt, permutation importance and Jackknife tests were used to evaluate individual variable contributions. For Random Forest and BRT, importance was determined based on mean decrease in accuracy and gain, respectively. In GLM, standardized regression coefficients and p-values were examined. | | |
|  |  |  |  | | Model selection / Model averaging / Ensembles |  | | · Model selection strategy:Model selection was based on predictive performance rather than information-theoretic criteria | | |
|  |  |  |  |  |  |  | | · Method for model averaging: algorithtic average for 13 GCMs | | |
|  |  |  |  |  |  |  | | · Ensemble method: 13 GCMs are averaged for future climate prediction | | |
|  |  |  |  | | Non-independence correction/analyses |  | | · Method for addressing spatial autocorrelation in residuals: No explicit spatial autocorrelation test was conducted. However, spatial thinning of occurrence records (1 km grid) was applied to reduce spatial clustering and mitigate autocorrelation effects in model inputs. Additionally, 5-fold cross-validation with spatially independent folds was used to avoid spatial overfitting in evaluation | | |
|  |  |  |  |  |  |  | | · Method for addressing temporal autocorrelation in residuals:Not applicable. The study is based on long-term climate averages (1970–2000 baseline and 2090s projections) and does not involve time-series data or repeated measures over time | | |
|  |  |  |  |  |  |  | | · Method to account for nested data:Not applicable. All models were trained using flat presence-only data with no nested or hierarchical structure | | |
|  |  |  |  |  | Threshold selection |  |  | · Details on threshold selection:The threshold for converting continuous habitat suitability into binary predictions (suitable/unsuitable) was set at 0.5, which is the default and widely used threshold | | |
|  | | **A**ssessment |  | | Performance statistics |  | | · Performance statistics estimated on training data:Performance on training data was not reported separately, as the evaluation focused on model generalization via cross-validation. All performance metrics (AUC, TSS, Kappa) were calculated on validation partitions to avoid overfitting. | | |
|  |  |  |  | |  |  |  | · Performance statistics estimated on validation data (from data partitionin:Model performance was assessed using 5-fold cross-validation . Evaluation metrics included AUC, TSS, and Cohen’s Kappa. | | |
|  |  |  |  | |  |  |  | · Performance statistics estimated on test (truly independent) data: NA | | |
|  |  |  |  | | Plausibility check |  | | · Response plots: Univariate response curves were generated for each predictor in MaxEnt, RF, and BRT models to visualize the effect of individual variables | | |
|  |  |  |  |  |  |  | | · Expert judgements: Predicted suitability maps were visually evaluated by all authors | | |
|  |  | **P**rediction |  |  | Prediction output |  |  | · Prediction unit:Habitat suitability scores (logistic output from MaxEnt) are categorized into 4 levels and binary (0.5 as the threshold), depending on analysis purposes. | | |
|  |  |  |  |  |  |  | | · Post-processing:Final predictions were clipped to the Wuyi Mountains region (GMBA polygon) | | |
|  |  |  |  |  | Uncertainty quantification |  | | · Algorithmic uncertainty:Addressed by comparing outputs from four modeling algorithms (MaxEnt, RF, BRT, GLM). | | |
|  |  |  |  |  |  |  | | · Uncertainty in input data:Occurrence data spatially thinned (1 km) to reduce location bias. Environmental layers harmonized in resolution. Static assumptions for soil and human footprint layers acknowledged | | |
|  |  |  |  |  |  |  | | · Effect of parameter uncertainty, error propagation:Not quantified directly. However, replicate model runs and response curve examination helped assess the robustness of model behavior | | |
|  |  |  |  |  |  |  | | · Uncertainty in scenarios: Future projections under two climate scenarios (SSP126 and SSP585) using ensemble means from 13 GCMs (CMIP6) | | |
|  |  |  |  |  |  |  | | · Visualisation/treatment of novel environments:Novel environmental conditions were visualized using Distance to Centroid Index (DI) and Area of Applicability (AOA) analyses to identify extrapolation risks and mask unreliable predictions | | |
